# Flow Cytometric Assessment of the Viability and Functionality of Uterine Polymorphonuclear Leukocytes in Postpartum Dairy Cows

**DOI:** 10.3390/ani11041081

**Published:** 2021-04-10

**Authors:** Leen Lietaer, Kristel Demeyere, Stijn Heirbaut, Evelyne Meyer, Geert Opsomer, Osvaldo Bogado Pascottini

**Affiliations:** 1 Department of Reproduction, Obstetrics and Herd Health, Faculty of Veterinary Medicine, Ghent University, 9820 Merelbeke, Belgium; leen.lietaer@ugent.be (L.L.); geert.opsomer@ugent.be (G.O.); 2 Department of Pharmacology, Toxicology and Biochemistry, Faculty of Veterinary Medicine, Ghent University, 9820 Merelbeke, Belgium; kristel.demeyere@ugent.be (K.D.); evelyne.meyer@ugent.be (E.M.); 3 Laboratory for Animal Nutrition and Animal Product Quality, Department of Animal Sciences and Aquatic Ecology, Faculty of Bioscience Engineering, Ghent University, 9000 Ghent, Belgium; stijn.heirbaut@ugent.be; 4 Laboratory for Veterinary Physiology and Biochemistry, Department of Veterinary Sciences, University of Antwerp, 2610 Wilrijk, Belgium

**Keywords:** bovine, neutrophil, flow cytometry, reproductive tract, endometritis

## Abstract

**Simple Summary:**

Dairy cows experience immune suppression around calving, which can result in the development of uterine diseases. White blood cells, and more specifically polymorphonuclear leukocytes (PMN), are important components of the immune system. Although it is regarded as a common research approach to study PMN isolated from blood, it would be interesting to know more about the viability and functionality of uterine PMN. We developed a method to isolate PMN from the bovine postpartum uterus to perform viability and functionality tests. We also evaluated whether we could identify uterine PMN using a specific antibody. Uterine cells were recovered using an adapted medical brush, and PMN were successfully isolated and identified. The percentage of viable intra-uterine PMN in postpartum cows (9–37 days in milk) roughly ranged from 10 to 80%, indicating that the viability of the uterine PMN is highly dynamic. We could also identify PMN that ingested labeled particles, which lets us conclude that uterine PMN are functional. Using the presently described methods, further research can be performed to unravel the role of uterine PMN viability and functionality in bovine uterine health.

**Abstract:**

Postpartum dairy cows experience impaired peripheral polymorphonuclear leukocyte (PMN) functionality, which has been associated with reproductive tract inflammatory diseases. However, it has not been elucidated yet whether endometrial PMN functionality is (equally) impaired. We developed a method for endometrial PMN isolation and flow cytometric assessment of their viability and functionality. We also evaluated PMN immunolabeling, using a specific bovine granulocyte marker, CH138A. Blood and endometrial cytobrush samples were collected in duplicate from seventeen clinically healthy Holstein-Friesian cows between 9 and 37 days in milk. The proportion of viable, apoptotic, and necrotic PMN in endometrial samples roughly ranged from 10 to 80%, indicating highly dynamic endometrial PMN populations in the postpartum uteri. Endometrial PMN functionality testing revealed that PMN immunolabeling increased the accuracy, although this protocol might influence the median fluorescence intensity of the sample. Phagocytosis seemed the most stable and reliable endometrial PMN function and could be assessed satisfactorily without prior CH138A immunolabeling. However, the interpretation of oxidative burst and intracellular proteolysis tests remains challenging. The correlation between peripheral and endometrial PMN functionality was poor. Further research is warranted to unravel the role of uterine PMN viability and functionality in bovine uterine health.

## 1. Introduction

Periparturient dairy cows experience an increased energy demand due to steady growth of the foetus, commencement of lactation, and immune system activation [1,2,3]. Simultaneously, feed intake is decreased, resulting in negative energy balance (NEB) and consequent fat mobilization accompanied by increased concentrations of circulating non-esterified fatty acids (NEFA) and ketone bodies (e.g., β-hydroxybutyrate, BHB) [1,4,5]. Excessive lipolysis and the limited availability of amino acids and glucogenic compounds may result in maladaptation to this NEB. High circulating concentrations of NEFA, BHB and the acute-phase protein haptoglobin are risk factors for reproductive tract inflammatory diseases, which affect almost 50% of postpartum dairy cows [6,7,8]. Reproductive tract inflammatory diseases interfere with key reproductive events such as folliculogenesis, sperm function, and (early) embryo development and implantation, resulting in an overall reduction of reproductive performance [9,10,11].

Polymorphonuclear leukocytes (PMN) are the most extensively studied component of the innate immune response against invading pathogens in the bovine genital tract postpartum [12,13]. Besides their role in the first-line defense, PMN are important for maintaining homeostasis, tissue remodeling, and wound healing [13,14]. Nevertheless, peripheral PMN function is impaired postpartum [15,16], which partially explains the increased risk for postpartum reproductive tract inflammatory diseases in cows with excessive NEB and systemic inflammation [8,17].

After calving, fresh PMN are rapidly recruited from the bone marrow, and migrate via the bloodstream towards the uterine lumen [12,18,19]. On the one hand, a swift and well-regulated PMN uterine migration and subsequent local function are required for the timely and efficient elimination of pathogens, preventing reproductive disease in the early postpartum period [20,21]. On the other hand, an increased abundance of PMN (i.e., >5%) within the uterine lumen after the completion of the involution process (in the fifth week postpartum), is associated with subfertility [22,23]. Calculating the proportion of PMN in endometrial cytology smears is a simple technique commonly performed to assess the uterine inflammatory status in dairy cows [22,24]. Nevertheless, neither the viability, nor the functionality of endometrial PMN can be assessed using conventional endometrial cytology.

Traditionally, PMN function has been tested on PMN isolated from blood. After their isolation and in vitro stimulation with either bacteria or chemokines, flow cytometric assessment of their per-cell capacity of phagocytosis or production of reactive oxygen species (ROS) is routinely performed [25,26,27]. Though, to date, it remains unclear whether the outcome of in vitro function tests of circulating PMN correlates with the functionality of (activated) PMN at their target site.

The present study had three main objectives: firstly, to develop a method for the isolation of endometrial PMN and the subsequent evaluation of their viability; secondly, to flow cytometrically assess the endometrial PMN functions—oxidative burst, phagocytosis, and intracellular proteolysis, adapting validated protocols for blood PMN [27,28]; thirdly, to evaluate the correlation between the functionality of endometrial PMN and their concurrently isolated blood counterparts in postpartum dairy cows. While blood PMN can easily be differentiated and gated based on their characteristic morphometrical size (forward scatter, FSC) and granularity (side scatter, SSC), the differentiation of endometrial PMN from debris and epithelial cells is challenging. Therefore, we included some extra intra- and inter-assay validation steps to evaluate the added value of the simultaneous immunolabeling with a specific bovine granulocyte marker, CH138A [29,30,31]. Based on these objectives, we hypothesized that PMN isolated from the uterus of postpartum dairy cows are viable and functional, and that their functionality is positively correlated with the functionality of circulating PMN.

## 2. Materials and Methods

### 2.1. Ethical Statement

All animal handling and sampling procedures were approved by the ILVO (Flanders Research Institute for Agriculture, Fisheries and Food) animal ethics committee (EC 2018/329, Melle, Belgium).

### 2.2. Animals and Management

The present study was carried out at the experimental dairy farm of ILVO (Melle, Belgium) between February and October 2020. During the time frame of the study, the per farm average daily milk yield was 31.5 kg/cow, with a mean of 4.3% fat and 3.6% protein (test-day recording, Cattle Improvement Co-operative CRV, Arnhem, the Netherlands). The transition cow management was as follows: pregnant dry cows were housed in a free stall barn and moved to calving pens when imminent calving indicators were observed (e.g., udder distension, teat filling, pelvic ligament relaxation) or 3 days before the expected calving date. Three days after calving, cows were moved to a free stall lactating pen, where they were fed a totally mixed ration from individually assigned automated feed bins (Insentec B.V., Marknesse, The Netherlands), and were milked twice daily in a parlour.

The sample size for this experiment consisted of seventeen randomly selected Holstein-Friesian cows from 9 to 37 days in milk (DIM), but some cows were sampled more than once at different DIM. All included cows were without signs of clinical disease (e.g., no fever) at the moment of sampling, and were multiparous, ranging from parity 2–6. Their prepartum body condition score was (3.3 ± 0.6) [32].

We performed two experiments in different cohorts (first experiment between February and July, second experiment between August and October). Cows were randomly included in either experiment. Fifteen blood and endometrial samples were collected for Experiment 1, and 11 blood and endometrial samples were collected for Experiment 2. Sample sizes were calculated to enable the detection of a fairly high correlation (0.7–0.8) between two variables at a significance level of 0.05 (power = 0.8), appropriate for a validation study [33]. Blood and endometrial samples were collected in the morning (between 9 h and 10 h).

### 2.3. Experiment 1: Assessment of PMN Viability in Blood and Endometrial Samples

We aimed to validate a flow cytometric test to evaluate blood and endometrial PMN viability. To do so, blood and endometrial samples were collected in duplicate (A and B). Samples were processed in parallel, to assess the repeatability of the assay.

#### 2.3.1. Blood Sample Collection and PMN Isolation

Blood samples were collected in duplicate from the coccygeal vessels, using 20-gauge blood collection needles (BD Vacutainer, PrecisionGlide, Becton Dickinson, Plymouth, UK) into sterile glass tubes containing 1.5 mL acid citrate dextrose-A (ACD-A; 22 g/L trisodium citrate, 8 g/L citric acid, and 24.5 g/L dextrose; BD Vacutainer, Becton Dickinson, Plymouth, UK). After collection, blood tubes were gently inverted 10 times, enabling adequate mixing, and placed in ice for transportation. The PMN isolation protocol started within 2 h after sampling. At room temperature, 8 mL of blood was diluted in 20 mL of a stock solution containing 1× phosphate buffered saline (PBS; Gibco/Thermo Fisher Scientific, Waltham, MA, USA) and 0.5 mM of ethylenediaminetetraacetic acid (EDTA) disodium salt (Sigma-Aldrich, Oakville, ON, Canada). The diluted blood was carefully layered on 8 mL of Ficoll-Paque PLUS (General Electric Healthcare Bio-Sciences AB, Uppsala, Sweden) and centrifuged at 340× *g* for 30 min at room temperature. The upper layers (plasma and buffy coat) were drawn off using a 10 mL serological pipette, and the erythrocytes were lysed by adding 30 mL of sterile water (water for injection, B. Braun, Melsungen, Germany) followed by gentle inversion for 45 s. To restore osmolarity, 15 mL of 3× concentrated PBS was added, mixing by gentle inversion. The samples were centrifuged at 4 °C at 220× *g* for 10 min. Next, the supernatant was removed, and the resultant pellet was washed with 500 µL of 1× PBS. The lysis steps (adding 30 mL of sterile water for 45 s and 15 mL of 3× concentrated PBS) were repeated and the samples were centrifuged again at 4 °C at 220× *g* for 10 min. After this, the supernatant was removed and the pellet was re-suspended in 1 mL of a stock solution containing RPMI medium 1640 (Gibco/Thermo Fisher Scientific, Waltham, MA, USA) and 0.1% bovine serum albumin (BSA, ≥96%, Sigma-Aldrich, Oakville, ON, Canada), hereafter referred to as RPMI-BSA. The concentration of PMN was assessed using a Bürker counting chamber, and the cell suspension was diluted to a final concentration of 5 × 10^6^ of PMN in RPMI-BSA.

#### 2.3.2. Endometrial Sample Collection and PMN Isolation

After the collection of the blood sample, the perineum of the cows was cleaned with fresh water and iodide soap, dried with paper towels, and disinfected with ethanol 70%. A double-guarded sterile cytobrush device (cytology brush equine, Minitube, Tiefenbach, Germany; or Puritan histobrush, Guilford, ME, USA, adapted to a stainless-steel artificial insemination gun and covered with a plastic sanitary sheath) was introduced into the vagina and manipulated through the cervix, under rectal guidance. Once the tip of the device reached the uterine body, the outer guard, or the sanitary sheath, was pulled back, and the cytobrush was exposed from the inner guard. The cytobrush was rotated 3 times against the dorsal wall of the uterine body with gentle pressure of the index finger through the rectum. The cytobrush was then retracted and removed from the vagina. Once outside the genital tract, the cytobrush was gently rolled onto a clean microscope slide which was then air-dried. The head of the cytobrush was then cut with scissors and placed in a 1.5 mL microcentrifuge tube containing 1 mL of RPMI-BSA supplemented with 0.18% K2EDTA (BD Vacutainer, Becton Dickinson, Plymouth, UK), hereafter referred to as RPMI-BSA-EDTA. A second, identical cytobrush sample was collected and processed as described above, and both samples were transported on ice to the laboratory. The PMN isolation protocol started within 2 h after sampling.

Cytology slides were stained with Diff-Quick (Speedy-Diff complete kit, Clin-Tech Ltd., Guildford, UK) and mounted with Eukitt (O. Kindler GmbH, Freiburg, Germany). Light microscopic evaluation was done at a magnification of ×40 (Kyowa Optical, Tokyo, Japan). A total of 300 nucleated cells were counted in randomly selected fields, and the PMN-to-other cell ratio was calculated. All specimens were counted by the same observer (trained veterinarian).

The microcentrifuge tubes containing 1 mL of RPMI-BSA-EDTA and the endometrial samples were vortexed for 1 min to dislodge cells from the cytobrush. The cytobrush was then gently rubbed against the border while removed from the vial. Samples were centrifuged at 4 °C at 376× *g* for 10 min. Then, supernatant was removed, and the pellet was washed for 45 s in 800 µL of sterile water (water for injection, B. Braun, Melsungen, Germany) to enable the lysis of the erythrocytes. To restore osmolarity, 400 µL of 3× PBS was added and gently mixed by pipetting up and down. The samples were again centrifuged at 4 °C at 376× *g* for 10 min, next the supernatant was removed, and the pellet was re-suspended with 2 mL of RPMI-BSA and filtered through a 40 µm mesh cell strainer (Falcon, Corning Life Sciences, Tewksbury, MA, USA). The concentration of PMN was assessed manually using a Bürker counting chamber (Marienfeld GmbH & Co. KG, Lauda-Königshofen, Germany), and the cell suspension was diluted to a final concentration of 5 × 10^6^ PMN in RPMI-BSA. Samples with lower concentrations of PMN were not diluted.

#### 2.3.3. Blood and Endometrial PMN Immunolabeling

Blood PMN and uterine cells were isolated as described above and labeled with a specific primary mouse anti-bovine IgM monoclonal granulocyte antibody, CH138A (Washington State University, Monoclonal Antibody Center, BOV2067, Pullman, WA, USA) [29,30,31]. Briefly, two 1.5 mL microcentrifuge tubes were filled with 1 × 10^6^ PMN suspended in 200 µL of in RPMI-BSA. In the first tube (V+), primary and secondary fluorescent labeling were applied, while in the second tube (V−) no antibodies were added (autofluorescence). All tubes were centrifuged at 10 °C at 376× *g* for 5 min and the pellet was resuspended in 100 µL flow cytometry staining buffer (FACS buffer; 1× PBS with 1% BSA and 0.073% EDTA). Cells were incubated in FACS buffer containing 1 µL of CH138A (final concentration of 10 μg/mL) (V+), or FACS buffer only (V−) for 30 min at 4 °C in the dark. After incubation, cells were washed with FACS buffer (200 µL, 376× *g*, 5 min, 10 °C). Next, cells were incubated for 15 min at 4 °C in the dark in 100 µL FACS buffer with a secondary antibody (goat anti-mouse IgM Alexa-647; final concentration of 4.8 µg/mL, A-21238, Molecular Probes, Invitrogen, Carlsbad, CA, USA) (V+) or in FACS buffer only (V−). After incubation, cells were washed twice in FACS buffer (200 µL, 376× *g*, 5 min, 10 °C). A total of 1 mL of Ca^++^-rich incubation buffer (10 mM HEPES, 140 mM NaCl, 5 mM CaCl_2_, pH 7.4) was prepared and mixed with 20 µL of Annexin-V-FLUOS (fluorescein; Roche Diagnostics GmbH, Mannheim, Germany) and 20 µL of propidium iodide (PI; final concentration of 1 µg/mL, Molecular Probes, Invitrogen, Belgium). After immunolabeling with CH138A, 100 µL of the Annexin-PI working solution was added to the cell pellet (V+), or 100 µL of FACS buffer (V−). Cells were incubated for 10 min at room temperature in the dark. Then, all samples were put on ice and protected from light until analysis.

#### 2.3.4. Sorting CH138A Positive Cells

To confirm the specificity of CH138A immunolabeling in endometrial PMN, we used 3 endometrial samples from 3 postpartum cows. Briefly, endometrial cytobrush samples were collected, and endometrial cells were isolated and CH138A labeled as described above. Then, PMN were purity-sorted, based on CH138A^+^ expression, using a BD FACS Aria III Cell Sorter (BD Biosciences, San Jose, CA, USA). Next, cytocentrifuge (Shandon Scientific, London, UK; 800 rpm for 10 min) slides were prepared and stained with Diff-Quick (Speedy-Diff complete kit, Clin-Tech Ltd., Guildford, UK) and mounted with Eukitt (O. Kindler GmbH, Freiburg, Germany). Light microscopic evaluation was done at magnification ×40 (Kyowa Optical, Tokyo, Japan). A total of 300 nucleated cells were counted in randomly selected fields. All samples had >85% PMN purity. Thus, CH138A PMN labeling was considered successful and we were able to continue with further experiments.

#### 2.3.5. Flow Cytometric Approach to Evaluate PMN Viability

**Figure 1:**
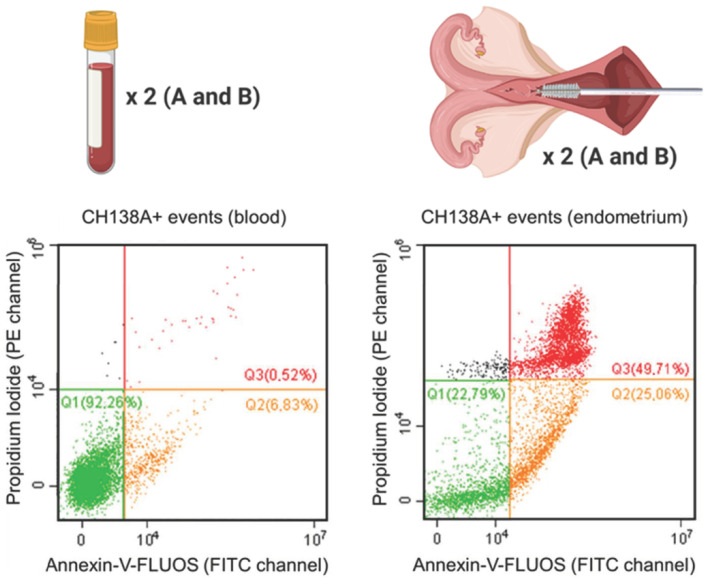
Viability of isolated blood and endometrial polymorphonuclear leukocytes (PMN) represented by Annexin-V-FLUOS (FITC channel) versus the propidium iodide (PI; PE channel) scatter plot. This figure shows the viable (Q1 = Annexin^−^/PI^−^), apoptotic (Q2 = Annexin^+^/PI^−^) and necrotic (Q3 = Annexin^+^/PI^+^) CH138A^+^ PMN in a representative blood and endometrial (70% PMN on endometrial cytology) sample from a Holstein-Friesian cow 21 days in milk. In total, 15 blood and endometrial samples were collected from Holstein-Friesian cows at 9–37 days in milk. Samples were processed in duplicate (A and B), with prior CH138A immunolabeling (CH138A = bovine granulocyte marker). The repeatability of the viability assessment was calculated between samples A and B in endometrium and blood.

Blood and endometrial samples were analyzed using a CytoFLEX 3-laser flow cytometer (Beckman Coulter Inc., Indianapolis, IN, USA). For all samples, the event recording threshold was set at 10,000 events in a high forward (FSC) × high side scatter (SSC) gate on FSC vs. SSC plots, or in a measuring time of 120 s (the acquisition velocity was 30–60 µL/min). Cell fluorescence was excited at 488 and 638 nm, and all fluorescent emissions were measured: CH138A fluorescent immunolabeling was measured using the emission filters of 660 ± 10 nm (APC channel), PI was measured using the 585 ± 21 nm filter (PE channel), and Annexin-V-FLUOS was measured with the 525 ± 20 nm filter (FITC channel) (Supplementary Table S1). All acquired data were processed using CytExpert software (v2.0.0.153, Beckman Coulter, Inc., Brea, CA, USA). Compensation was applied in multicolor setups. Blood PMN were identified based on their characteristic FSC and SSC values (PMN gate, Supplementary Figures S1 and S2). For endometrial samples, all events were included, only excluding a debris fraction on the FSC vs. SSC plot (cells excluding debris; Supplementary Figures S1 and S3). Next, the single cell population was gated on an FSC-A vs. FSC-H scatter plot to exclude aggregates (Supplementary Figure S1). Using tricolor labeling for PMN viability (CH138A × Alexa Fluor 647/Annexin-V-FLUOS/PI), three populations can be differentiated: viable CH138A^+^ PMN (Annexin^−^/PI^−^), apoptotic CH138A^+^ PMN (Annexin^+^/PI^−^), and necrotic CH138A^+^ PMN (Annexin^+^/PI^+^) (Figure 1).

### 2.4. Experiment 2: Blood and Endometrial PMN Functionality Tests

**Figure 2:**
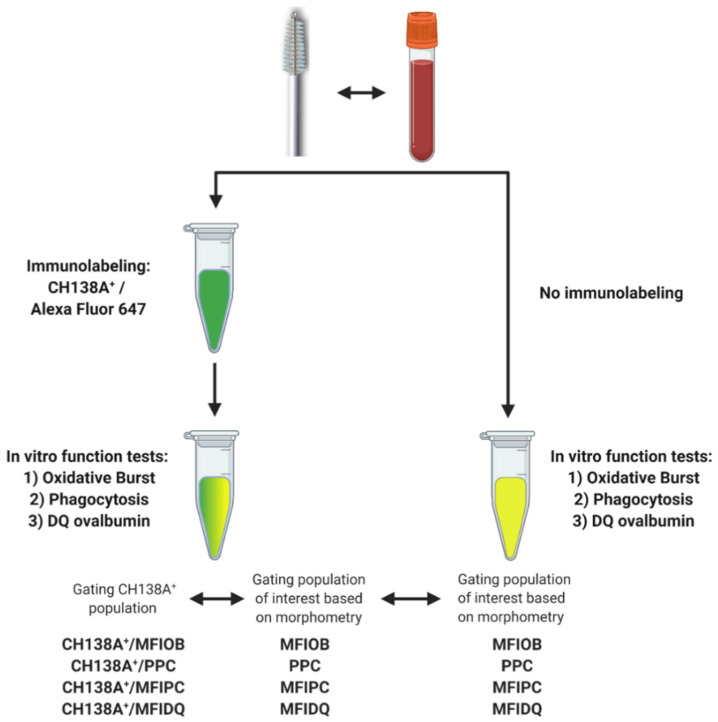
Experimental setup for blood and endometrial polymorphonuclear leukocyte (PMN) functionality tests assessing PMN oxidative burst (OB), phagocytosis (PC), and intracellular proteolytic degradation by DQ-ovalbumin (DQ-ova). Blood and endometrial samples (*n* = 11) were collected from Holstein-Friesian cows at 9–37 days in milk. The PMN functionality tests were performed in duplicate, once with prior CH138A immunolabeling and once without CH138A immunolabeling (CH138A = bovine granulocyte marker). In samples with CH138A immunolabeling, we compared the functionality outcome intra-assay of CH138A^+^ events versus a gating strategy solely based on the morphometric characteristics high forward scatter (FSC) vs. high side scatter (SSC). An inter-assay comparison was made between immunolabeled PMN and non-immunolabeled PMN, gating PMN solely based on the morphometric characteristics high FSC and high SSC. Figure created with BioRender.com (accessed on 10 February 2021).

We tested a flow cytometric assay to evaluate endometrial PMN functionality. PMN functionality tests have already been performed in blood, so blood samples were processed in parallel and were used as a positive control [27,28]. We also evaluated the use of CH138A immunolabeling to simultaneously identify PMN and assess their functions. The collection of blood and endometrial samples and the PMN isolation were performed in duplicate as described above. However, based on the high repeatability of duplicate samples obtained in Experiment 1 (see Section 3) and because we aimed for a higher (a single cytobrush sample will not provide sufficient PMN for all the tests) and equal yield in all PMN suspensions (duplicate cytobrush may contain different PMN numbers) for the validation of the functionality tests, suspensions from Samples A and B were combined in a single microcentrifuge tube after isolation. The PMN functionality tests were performed in duplicate, one with and one without CH138A immunolabeling (Figure 2, Supplementary Figures S4 and S5), each of them with their respective control group (for autofluorescence).

#### 2.4.1. Oxidative Burst

For every sample (blood and endometrium), two 1.5 mL microcentrifuge tubes were filled with 1 × 10^6^ PMN suspended in 200 µL of in RPMI-BSA, and 2 µL of H2DCFDA (final concentration of 10 µM; 2′,7′-dichlorodihydrofluorescein diacetate, Life Technologies Corporation, Eugene, OR, USA) was added to both tubes (OB+ and OB−). Next, cells were incubated in the dark at 38.5 °C under gentle agitation. After 15 min of incubation, 200 µL of phorbol 12-myristate 13-acetate (PMA; Sigma-Aldrich, Winston Park, Oakville, ON, Canada; 200 ng/mL in RPMI-BSA) was supplemented to the first tube (OB+), and 200 µL of RPMI-BSA was supplemented in the second tube (OB−). Cells were incubated in the dark at 38.5 °C under gentle agitation for another 15 min. After incubation, cells were washed and diluted in RPMI-BSA buffer (200 µL, 376× *g*, 5 min, 10 °C), put on ice and protected from light until analysis.

#### 2.4.2. Phagocytosis

A stock solution of zymosan-activated serum (ZAS) was prepared beforehand by incubating 10 mL of pooled blood serum from healthy cows with 100 mg of Zymosan A from *Saccharomyces cerevisiae* (Sigma-Aldrich, Winston Park, Oakville, ON, Canada) at 37 °C under gentle agitation for 60 min. After incubation, the activated serum was centrifuged at 390× *g* for 10 min, aliquoted and stored at −20 °C.

For every sample (blood and endometrium), two 1.5 mL microcentrifuge tubes were filled with 1 × 10^6^ PMN suspended in 200 µL of RPMI-BSA, and 50 µL of ZAS was added to both tubes (PC+ and PC−). In the first tube (PC+), 1 µL fluorescently labeled 1 µm beads (FluoSpheres carboxylate, yellow-green (505/515), Life Technologies Corporation, Eugene, OR, USA) was added per 1 × 10^6^ PMN, while no FluoSpheres were supplemented in the second tube (PC−, autofluorescence). Next, cells were incubated in the dark at 38.5 °C under gentle agitation for 30 min. After incubation, cells were washed and diluted in RPMI-BSA buffer (200 µL, 376 × *g*, 5 min, 10 °C), put on ice and protected from light until analysis.

#### 2.4.3. Intracellular Proteolytic Degradation by DQ Ovalbumin

For every sample (blood and endometrium), two 1.5 mL microcentrifuge tubes were filled with 2 × 10^6^ PMN suspended in 400 µL of in RPMI-BSA. Next, all tubes were centrifuged at 10 °C at 376× *g* for 5 min and the pellet was re-suspended in 120 µL of RPMI-BSA. In the first tube (DQ+), 10 µL of DQ ovalbumin (10 μg/mL, Life Technologies Corporation, Eugene, OR, USA) was applied, while in the second tube (DQ−) no DQ ovalbumin was supplemented for the assessment of autofluorescence. Cells were then incubated in the dark at 38.5 °C under gentle agitation for 45 min [27]. After incubation, cells were washed and diluted in RPMI-BSA buffer (200 µL, 376× *g*, 5 min, 10 °C), put on ice and protected from light until analysis.

#### 2.4.4. Flow Cytometric Approach to Evaluate PMN Functionality

Samples were evaluated as described for Experiment 1 with some modifications. Briefly, the fluorescence emitted in the oxidative burst (OB), phagocytosis (PC), and DQ ovalbumin (DQ-ova) assays were measured with the 525 ± 20 nm filter (FITC channel) (Supplementary Table S1). First, a high FSC × high SSC subpopulation was gated (Supplementary Figures S4 and S5). Second, the single cell population was gated on an FSC-A vs. FSC-H scatter plot to exclude aggregates. For the samples without CH138A immunolabeling, blood and endometrial PMN were identified solely based on morphometry (high FSC and high SSC values; Figure 2, Supplementary Figures S4 and S5). Within this gate, the percentage of cells that performs PC (PPC) was calculated based on the positive green signal of the FluoSpheres. To evaluate OB (MFIOB), PC (MFIPC), or proteolytic degradation via DQ-ova (MFIDQ), the difference between the median fluorescence intensity (MFI) of positive cells and the MFI of the respective autofluorescence control was calculated (Supplementary Figure S6). For the samples with CH138A immunolabeling, two gating strategies were applied (Figure 2, Supplementary Figures S4 and S5): one as described above as for samples without CH138A immunolabeling, and another one in which CH138A^+^ events were identified and gated before measuring PPC, MFIOB, MFIPC and MFIDQ (in respect to their autofluorescence controls).

#### 2.4.5. Intra-Assay Validation Approach

We evaluated if PMN immunolabeling using CH138A is necessary to assess the endometrial PMN function. To do so, in samples with CH138A immunolabeling, we compared the functionality outcome intra-assay of CH138A^+^ events versus a gating strategy solely based on the morphometrical characteristics high FSC vs. high SSC (Figure 2, Figures S4 and S5). Briefly, samples were immunolabeled with CH138A as described for Experiment 1. After immunolabeling with CH138A, all tubes were centrifuged at 10 °C at 376× *g* for 5 min and the pellet was resuspended in 200 µL RPMI-BSA. PMN function tests were then performed as described above and results were compared intra-assay.

#### 2.4.6. Inter-Assay Validation Approach

We evaluated the effect of CH138A immunolabeling on PMN functionality inter-assay. PMN function tests were performed as described above. The PMN gating strategy was solely based on the morphometrical characteristics high FSC vs. high SSC and results were compared inter-assay between samples with versus without CH138A immunolabeling (Figure 2).

### 2.5. Statistics

Data were exported and processed using the R language for statistical programming [34] (v3.6.0). Descriptive statistics were calculated for all cows and flow cytometry parameters. The normality of the distributions was verified using histograms and Shapiro–Wilk tests (*p* ≥ 0.05 is normally distributed). Lin’s concordance correlation coefficient (CCC) analysis was calculated to assess the degree of agreement between the different corresponding or repeated measurements and flow cytometric variables. *p*-values for the CCC were calculated by the corresponding Pearson (parametric data) or Spearman (non-parametric data) correlation tests. Furthermore, pairwise *t*-tests (parametric data) or Wilcoxon signed rank sum tests (non-parametric data), and corresponding *p*-values were calculated for all functionality outcomes for the intra- and inter-assay validation experiments to evaluate potential differences caused by immunolabeling and/or gating strategies. Results were similarly assessed (CCC and pairwise *t*-tests) between PMN functionality tests in blood and endometrium. Analyses and visualization were done using the R-packages Rcmdr [35] (v2.7.1), DescTools [36] (v0.99.40), ggplot2 [37] (v3.3.3), and ggpubr [38] (v0.4.0). Differences were considered significant at *p* < 0.05.

## 3. Results

### 3.1. PMN Isolation and Immunolabeling

The number of PMN isolated from a single blood sample (*n* = 41) was 23.1 ± 11.8 × 10^6^ (mean ± standard deviation (SD); range 6 × 10^6^–60 × 10^6^). The number of PMN isolated from a single endometrial cytobrush sample (*n* = 41) was 7.4 ± 13.1 × 10^6^ (range 4 × 10^5^–76 × 10^6^). The proportion of CH138A^+^ events in endometrial cell suspensions (*n* = 41) was 28 ± 20.5% (range 1–68%), which was positively correlated (CCC = 0.7; confidence interval (CI) = 0.53–0.82; *p* < 0.001) with the proportion of PMN identified in endometrial cytology samples (*n* = 41; 27 ± 28%; range 0–95%).

For 15 endometrial samples (Table 1), a positive correlation was found between duplicate samples (A and B) for the proportion of PMN in endometrial cytology (CCC = 0.97; *p* < 0.001) and for the proportion of CH138A^+^ events in endometrial cell suspensions (CCC = 0.85; *p* < 0.001).

### 3.2. PMN Viability

Descriptive statistics and reproducibility (CCC tests) of the PMN viability assessment in blood and endometrium are presented in Table 1. After PMN isolation from the blood, the proportion of viable, apoptotic, and necrotic PMN in the samples was 91.9 ± 5.2% (range 80.3–99.1%), 7.1 ± 5.1% (range 0.3–18.9%), and 0.7 ± 0.3% (range 0.2–1.2%), respectively. In endometrial PMN, the proportion of viable, apoptotic, and necrotic PMN was 38.8 ± 17.3% (range 10.9–75.7%), 23.6 ± 16.9% (range 4.6–63.1%), and 33.5 ± 20.9% (range 6.1–70.6%), respectively. All viability outcomes were positively correlated between the duplicate samples (A and B) in blood and endometrium (CCC range 0.67–0.86 in blood, and 0.68–0.95 in endometrium; *p* < 0.01).

### 3.3. Blood and Endometrial PMN Functionality Tests

#### 3.3.1. Intra-Assay Validation Approach

Descriptive statistics of the function tests OB, PC, and DQ-ova in CH138A immunolabeled blood and endometrial PMN are shown in Table 2. In blood, all functionality outcomes were positively correlated between CH138A^+^ events and events gated solely based on morphometrical characteristics (high FSC and high SSC) (CCC range 0.7–0.97; *p* < 0.021). However, all functionality outcomes in blood were higher in CH138A^+^ events than in events gated solely based on morphometrical characteristics (*p* < 0.002).

For endometrial PMN, the PPC and the MFIPC of CH138A^+^ events was positively correlated with PPC and MFIPC of events gated solely based on morphometrical characteristics (CCC = 0.65 (PPC) and 0.86 (MFIPC); *p* < 0.001 for both). PPC and MFIPC were higher in CH138A^+^ events than in events gated solely based on morphometrical characteristics (*p* = 0.005 and 0.03, respectively). Conversely, the MFIOB and MFIDQ were not significantly correlated between CH138A^+^ events versus events gated solely based on morphometrical characteristics (CCC = 0.87 (MFIOB) and 0.41 (MFIDQ), *p* = 0.07 and 0.15, respectively). Interestingly, the MFIOB and MFIDQ of CH138A^+^ events were not different from the MFIOB and MFIDQ of events gated solely based on morphometrical characteristics (*p* = 0.13 and 0.25 for MFIOB and MFIDQ, respectively).

#### 3.3.2. Inter-Assay Validation Approach

Descriptive statistics of the function tests OB, PC, and DQ-ova in CH138A immunolabeled versus non-immunolabeled blood and endometrial PMN are shown in Table 3. For blood, PPC and MFIOB were positively correlated between CH138A immunolabeled versus non-immunolabeled samples (CCC = 0.57 (PPC) and 0.69 (MFIOB), *p* = 0.03 and 0.007, respectively). CCC between CH138A immunolabeled versus non-immunolabeled samples for MFIPC in blood was 0.2 (*p* = 0.06), while for MFIDQ it was 0.19 (*p* = 0.001). Functionality outcomes PPC, MFIPC, and MFIDQ in blood were significantly higher for CH138A immunolabeled versus non-immunolabeled samples (*p* < 0.002), while MFIOB was higher in non-immunolabeled samples (*p* = 0.01). For endometrial PMN, PPC and MFIPC were positively correlated between CH138A immunolabeled versus non-immunolabeled samples (CCC = 0.80 (PPC) and 0.61 (MFIPC), *p* = 0.02 for both). CCC between CH138A immunolabeled versus non-immunolabeled samples for MFIOB in endometrium was 0.43 (*p* = 0.07) and for MFIDQ in endometrium was 0.74 (*p* = 0.17). Functionality outcomes between immunolabeled and non-immunolabeled samples were not different in endometrial samples (*p* > 0.06).

### 3.4. Blood Versus Endometrial PMN Functionality

The correlations between blood and endometrial PMN functionalities are shown in Table 4. The CCC between blood and endometrial PMN functions were positive (CCC < 0.1), but not significant for PPC (*p* = 0.34), for MFIPC *(p* = 0.61), and for MFIOB (*p* = 0.18). However, there was a significant (*p* = 0.005), negative correlation (CCC = −0.01) between blood and endometrial MFIDQ. Functionality outcomes PPC, MFIPC, and MFIOB in non-immunolabeled samples were significantly higher in blood versus endometrium (*p* < 0.004), while MFIDQ was higher in endometrium (*p* = 0.02).

## 4. Discussion

Circulating PMN function is impaired during the transition period between late pregnancy and early lactation in dairy cows [15,16]. However, it has not been elucidated yet whether an impaired peripheral PMN functionality implies an (equally) impaired functionality of PMN in the uterus. In the present study, we first tested a straightforward method for the isolation of endometrial PMN from postpartum dairy cows and subsequently evaluated their viability. A high correlation was observed between duplicate cytobrush samples in their isolated endometrial PMN viability. Secondly, we described different protocols for endometrial PMN functionality testing, OB, PC and DQ-ova, and compared these with and without prior CH138A immunolabeling. Overall, PMN immunolabeling increased the accuracy of the functionality assays. However, results of most functionality assays, when based on morphometrical characteristics (i.e., high FSC and high SSC) highly correlated with those of the CH138A immunolabeling. Thirdly, in both datasets, blood and endometrial PMN functionality were poorly correlated.

When interpreting these data, a major finding was that the number of PMN isolated from the highest yielding endometrial cytobrush was almost 200 times greater than the number isolated from the lowest yielding sample. In marked contrast, in blood there was only a 10-fold difference between the highest and lowest yielding samples. This indicates that PMN are highly dynamic during the early postpartum period in dairy cows, influenced by both the number of days postpartum when samples are collected and the uterine health status [20,21,39,40]. Moreover, while flow cytometrical differentiation of blood PMN based on their morphometrical characteristic size (FSC) and granularity (SSC) is straightforward, the differentiation of PMN from the high number of debris and epithelial cells in endometrial samples is challenging, as also reported by our group for milk PMN [31]. Indeed, cell characteristics are expected to be severely altered by apoptosis and necrosis [41,42,43,44]. Therefore, we immunolabeled endometrial PMN, using a specific bovine granulocyte marker, CH138A, as also described for milk PMN by our group [29,30,31]. The specificity of the CH138A immunolabeling was high, resulting in >85% of PMN purity in sorted cells based on CH138A^+^ expression. This validation was essential since aspecific antibody binding to Fc-receptors of leukocytes is frequently reported in flow cytometrical assays [45]. Although we did not apply isotype control antibodies for this study, the usefulness and interpretation of isotype control antibodies is still unclear [45,46,47,48]. In this regard, we hypothesize that the complexity of the uterine environment would result in an intricate background signal, making the isotype control experiment highly unreliable. For instance, the (pro-inflammatory) environment of the postpartum uterus is rich in immunoglobulins which may compete with the Fc receptors, minimizing the possibility for aspecific binding of the CH138A × Alexa Fluor 647 [18,49]. Notably, the proportion of CH138A^+^ events in endometrial cell suspensions highly correlated with the proportion of PMN in endometrial cytology samples. The latter suggests that CH138A is a valuable marker for the correct identification of endometrial PMN.

The proportions of viable, apoptotic, and necrotic PMN isolated from endometrial samples all roughly ranged from 10 to 80%. Thus, not only is the proportion of PMN numbers highly dynamic in the uterus of postpartum cows, but also their life cycle substantially fluctuated. Hence, the multicolor flow cytometric protocol for endometrial PMN viability assessment described here provides an elegant tool aiding to understand the biorhythm of these innate immune cells within the uterine lumen. In future experiments, it will be interesting to examine associations between endometrial PMN viability at different days postpartum, including the eventual establishment of uterine disease.

For the second objective, we compared our two methodological approaches to study the endometrial PMN functionality in postpartum cows. We CH138A immunolabeled PMN prior to each functionality test and compared the functionality outcomes of CH138A^+^ events with those of the events gated solely based on the morphometrical characteristics (high FSC vs. high SSC). Although PPC and MFIPC were highly correlated between both events, results were systematically higher in the CH138A^+^ events. This suggests that there is no complete agreement between both populations, so that some of the non-PMN events (i.e., other cell types and debris) or (non-responsive) CH138A negative PMN end up in the high FSC × high SSC gate. Because function tests are traditionally performed in blood PMN without prior immunolabeling, blood samples were processed in parallel, to confirm our findings. Similar to endometrial samples, function tests of peripheral PMN highly correlated between CH138A^+^ events and events gated solely based on morphometrical characteristics. This was expected, since this PMN gating strategy is highly specific for blood [41]. However, all functionality outcomes in blood were again slightly higher in CH138A^+^ events than in events gated solely based on morphometrical characteristics. The latter suggests that also in blood a small number of non-PMN events (e.g., monocytes and mast cells), or (non-responsive) CH138A negative PMN end up in the high FSC × high SSC gate (Figure S2) [50]. Therefore, as stated before, CH138A immunolabeling resulted in a more accurate flow cytometric identification of both blood as well as endometrial PMN when assessing PMN functions.

Apart from the advantage of CH138A immunolabeling, the (extra) immunolabeling step is time consuming and labor intensive, and costly. Moreover, we hypothesize that it might also influence the MFI of the assays. Consequently, we evaluated the effect of the immunolabeling protocol on PMN functionality tests, by inter-assay comparison of the functionality outcomes between samples with and without CH138A immunolabeling. For endometrial samples, correlations for PPC and MFIPC between immunolabeled versus non-immunolabeled PMN were >0.6 and no significant differences were found. Moreover, our PPC results (15–60% of PMN phagocytosis in endometrium) are in line with those described by Brodzki et al. [51] (with values ranging from 20 to 45%), and slightly lower than results from Hussain and Daniel [52] and Mateus et al. [53] (both ranging from 40 to 90%). However, in blood we observed low correlations between PPC, MFIPC, and MFIDQ in immunolabeled versus non-immunolabeled PMN. In marked contrast, a good correlation was found between CH138A immunolabeled versus non-immunolabeled events for MFIOB, and remarkably higher MFI values were observed in non-immunolabeled PMN. The MFI of PC, OB, and DQ-ova assays can be influenced by cell type, cell activation, and time after sampling [27,54,55]. Consequently, we hypothesize that the extra centrifugation and incubation steps for the immunolabeling (pre-)activated blood PMN and resulted in higher PPC, MFIPC, and MFIDQ. Furthermore, it is plausible that the fluorescence intensity of OB, PC and DQ-ova is influenced by the fluorescence intensity of the CH138A × Alexa Fluor 647 itself. As for the endometrial samples, we hypothesize that the (pre-)activation effect of the immunolabeling might have been minimal, because cells originating from the postpartum uterine lumen might already have been activated [16,56]. In conclusion, we suggest that the immunolabeling protocol itself might influence the outcome of the PMN functionality tests. In addition, since endometrial PMN already might have been activated, function tests might be more difficult to interpret, especially OB and DQ-ova, which are probably a poor estimator for PMN functionality in endometrial samples.

For the third objective, we showed that the correlations between blood and endometrial PMN functionality were weak. Phagocytosis was significantly lower in endometrial versus blood PMN. This is in agreement with Zerbe et al. [57], who evaluated PMN functions after experimental mobilization of PMN towards the uterine lumen using Leukotriene B_4_ (LTB_4_). Also, Brodzki et al. [51] found higher PPC in circulating PMN than in endometrial PMN of healthy cows. In contrast to our results, Zerbe et al. [57] found that OB was not different between blood and uterine PMN. We can assume that the uterine environment and the time frame of 24 h between LTB_4_ injection and the retrieval of PMN were not completely identical with the physiological, non-experimentally induced uterine inflammatory response postpartum in our experiments.

In conclusion, we found that the population of endometrial PMN in clinically healthy postpartum dairy cows is highly dynamic, containing viable, apoptotic, and necrotic cells in variable proportions, which are probably depending on the days postpartum when samples were collected and uterine health status. Future studies, with the inclusion of a higher number of systematically collected samples, should be further investigated. Endometrial PMN are functional and capable of phagocytosis. However, the interpretation of OB and DQ-ova tests remains challenging. Due to the high correlation with immunolabeled PMN and because the CH138A itself (or its procedure) may interfere with the functionality tests, we hypothesize that flow cytometrical identification of endometrial PMN solely based on morphometrical characteristics might be a valid gating strategy for first-line evaluation of endometrial PMN phagocytosis. No evidence was found for correlations between PMN functions of circulating and endometrial PMN. Future research examining factors influencing endometrial PMN viability and phagocytosis will broaden our knowledge on PMN characteristics in the bovine uterus postpartum.

## Figures and Tables

**Table 1:** Descriptive statistics and reproducibility of identification of polymorphonuclear leukocytes (PMN) in endometrial samples (cytology and endometrial cell suspensions), and viability assessment of PMN isolated from blood and endometrium. The proportion of PMN identified in endometrial cytology samples (in %), and the proportion of CH138A^+^ events (labeled by a specific bovine granulocyte marker) in endometrial cell suspensions (in %), in duplicate (A and B) endometrial samples (*n* = 15). Flow cytometric evaluation of PMN viability (percentage viable, apoptotic, or necrotic) in duplicate (A and B) blood (*n* = 15) and endometrial samples (*n* = 15). All samples were collected from Holstein-Friesian cows from 9–37 days in milk. For flow cytometry, PMN were isolated, and immunolabeled using CH138A. Viability was assessed using bicolor labeling (Annexin-V-FLUOS/propidium iodide). Samples (A and B) were processed in parallel to assess the repeatability of the assay. Lin’s concordance correlation coefficient (CCC) and 95% confidence interval (CI) are reported with the *p*-value from the corresponding Pearson (parametric) or Spearman (non-parametric) test. Correlations were considered significant at *p* <0.05.

Variable	Sample	Mean ± SD (in %)	Range (in %)	CCC (95% CI)	*p*-Value
PMN in endometrial cytology	A	29.3 ± 30.7	0–80	0.97 (0.90–0.99)	<0.001
	B	28.7 ± 30.5	1–90		
CH138A^+^ cells in endometrium	A	31.7 ± 22.0	1.4–62.0	0.85 (0.62–0.95)	<0.001
	B	30.6 ± 21.5	5.7–68.1		
Viable PMN in blood	A	91.6 ± 5.82	80.3–99.1	0.86 (0.66–0.95)	<0.001
	B	92.1 ± 4.76	82.5–99.0		
Apoptotic PMN in blood	A	7.4 ± 5.7	0.3–18.9	0.86 (0.67–0.95)	<0.001
	B	6.9 ± 4.7	0.3–15.9		
Necrotic PMN in blood	A	0.7 ± 0.29	0.2–1.1	0.67 (0.26–0.87)	0.006
	B	0.7 ± 0.3	0.3–1.2		
Viable PMN in endometrium	A	40.0 ± 15.7	14.2–75.7	0.68 (0.25–0.88)	0.008
	B	37.6 ± 19.4	10.9–75.1		
Apoptotic PMN in endometrium	A	24.3 ± 17.6	4.6–59.9	0.95 (0.25–0.88)	<0.001
	B	23.0 ± 16.8	5.5–63.1		
Necrotic PMN in endometrium	A	32.2 ± 19.1	6.1–58.3	0.77 (0.44–0.92)	0.001
	B	34.7 ± 23.2	8.0–70.6		

**Table 2:** Flow cytometric evaluation of polymorphonuclear leukocyte (PMN) functionality via oxidative burst (OB), phagocytosis (PC), and intracellular proteolytic degradation by DQ ovalbumin (DQ-ova) by CH138A immunolabeling or morphometrical characteristics. Blood and endometrial samples (*n* = 11) were collected from Holstein-Friesian cows at 9–37 days in milk. All samples were CH138A immunolabeled (CH138A = specific bovine granulocyte marker) to identify PMN. The functionality outcomes of CH138A^+^ events versus a gating strategy solely based on morphometrical characteristics (high FSC and high SSC) were compared intra-assay. The functionality outcomes included: percentage of PMN that performs PC (PPC, in %), median fluorescence intensity (MFI) of PMN that displayed OB (MFIOB), PC (MFIPC), or DQ-ova (MFIDQ) relative to the MFI of the respective autofluorescence control. Lin’s concordance correlation coefficient (CCC) and 95% confidence interval (CI) are reported with the *p*-value from the corresponding Pearson (parametric) or Spearman (non-parametric) test. Correlations were considered significant at *p* < 0.05.

Variable	Gating Strategy	Outcome	Range	CCC (95% CI)	*p*-Value
PPC in blood (in %)	Morphometrics	83.82	69.36–92.03	0.70 (0.38–0.87)	0.021
	CH138A^+^	88.21	71.96–96.56		
MFIPC in blood	Morphometrics	1,780,888	483,827–4,043,406	0.89 (0.72–0.96)	<0.001
	CH138A^+^	2,156,790	505,748–4,659,214		
MFIOB in blood	Morphometrics	242,081	58,924–573,332	0.97 (0.93–0.99)	<0.001
	CH138A^+^	273,634	114,311–595,864		
MFIDQ in blood	Morphometrics	2519	1092–4631	0.94 (0.86–0.98)	<0.001
	CH138A^+^	2845	1223–5447		
PPC in endometrium (in %)	Morphometrics	40.35	15.44–70.15	0.65 (0.24–0.87)	<0.001
	CH138A^+^	49.92	20.02–91.76		
MFIPC in endometrium	Morphometrics	90,579	445–421,532	0.86 (0.56–0.96)	<0.001
	CH138A^+^	140,235	384–424,773		
MFIOB in endometrium	Morphometrics	−3073	−172,034–116,458	0.87 (0.71–0.95)	0.07
	CH138A^+^	14,643	−198,069–234,887		
MFIDQ in endometrium	Morphometrics	11,498	2392–19,693	0.41 (−0.25–0.81)	0.15
	CH138A^+^	8410	1982–18,191		

**Table 3:** Flow cytometric evaluation of polymorphonuclear leukocyte (PMN) functionality via oxidative burst (OB), phagocytosis (PC), and intracellular proteolytic degradation by DQ ovalbumin (DQ-ova) with or without prior CH138A immunolabeling. Blood and endometrial samples (*n* = 11) were collected from Holstein-Friesian cows at 9–37 days in milk. Samples were processed in duplicate, with or without prior CH138A immunolabeling. For both cases, the PMN gating strategy was solely based on morphometrical characteristics (high FSC and high SSC) and results were compared inter-assay. The functionality outcomes included: percentage of PMN that performs PC (PPC), median fluorescence intensity (MFI) of PMN that displayed OB (MFIOB), PC (MFIPC), or DQ-ova (MFIDQ) relative to the MFI of the respective autofluorescence control. CH138A = specific bovine granulocyte marker. Lin’s concordance correlation coefficient (CCC) and 95% confidence interval (CI) are reported with the *p*-value from the corresponding Pearson (parametric) or Spearman (non-parametric) test. Correlations were considered significant at *p* < 0.05.

Variable	CH138A	Outcome	Range	CCC (95% CI)	*p*-Value
PPC in blood (in %)	No	78.20	61.38–87.82	0.57 (0.20–0.80)	0.031
	Yes	83.82	69.36–92.03		
MFIPC in blood	No	1,116,458	450,894–1,841,761	0.20 (−0.19–0.53)	0.06
	Yes	1,780,888	483,827–4,043,406		
MFIOB in blood	No	373,274	85,403–1,013,163	0.69 (0.39–0.85)	0.007
	Yes	242,081	58,924–573,332		
MFIDQ in blood	No	974	404–1998	0.19 (0.01–0.35)	0.001
	Yes	2519	1092–4631		
PPC in endometrium (in %)	No	37.67	15.05–57.66	0.80 (0.44–0.94)	0.017
	Yes	40.35	15.44–70.15		
MFIPC in endometrium	No	38,637	375–244,415	0.61 (0.23–0.83)	0.021
	Yes	169,632	445–421,532		
MFIOB in endometrium	No	56,358	−13,006–477,606	0.43 (0–0.73)	0.067
	Yes	−3073	−172,034–116,458		
MFIDQ in endometrium	No	16,513	763–66,290	0.74 (0.11–0.95)	0.17
	Yes	11,498	2392–19,693		

**Table 4:** Flow cytometric evaluation of polymorphonuclear leukocyte (PMN) functionality in blood versus endometrium via oxidative burst (OB), phagocytosis (PC), and intracellular proteolytic degradation by DQ ovalbumin (DQ-ova). Blood and endometrial samples (*n* = 11) were collected from Holstein-Friesian cows at 9–37 days in milk. The functionality outcomes of endometrial PMN were related to blood PMN. Functionality measures included percentage of PMN that performs PC (PPC), median fluorescence intensity (MFI) of PMN that displayed OB (MFIOB), PC (MFIPC), or DQ-ova (MFIDQ) relative to the MFI of the respective autofluorescence control. Samples were not immunolabeled, and gating was performed based on morphometrical characteristics (high FSC and high SSC). Lin’s concordance correlation coefficient (CCC) and 95% confidence interval (CI) are reported with the *p*-value from the corresponding Pearson (parametric) or Spearman (non-parametric) test. Correlations were considered significant at *p* < 0.05.

Variable		Outcome	Range	CCC (95% CI)	*p*-Value
PPC (in %)	Blood	78.20	61.38–87.82	0.04 (−0.04–0.12)	0.34
	Endometrium	37.67	15.05–57.66		
MFIPC	Blood	1,116,458	450,894–1,841,761	0.01 (−0.03–0.04)	0.61
	Endometrium	38,637	375–244,415		
MFIOB	Blood	373,274	85,403–1,013,163	0.08 (−0.21–0.36)	0.18
	Endometrium	56,358	−13,006–477,606		
MFIDQ	Blood	974	404–1998	−0.01 (−0.03–0.00)	0.005
	Endometrium	16,513	763–66,290		

## Supplementary Materials

The following data are available online at https://www.mdpi.com/article/10.3390/ani11041081/s1, Table S1: Fluorescent dyes and their excitation and emission characteristics, Figure S1: Gating strategy for flow cytometric assessment of viability in bovine circulating (A) versus endometrial (B) polymorphonuclear leukocytes (PMN), Figure S2: Flow cytometric identification of blood polymorphonuclear leukocytes (PMN) using CH138A, Figure S3: Flow cytometric identification of endometrial polymorphonuclear leukocytes (PMN) using CH138A, Figure S4: Gating strategy for phagocytosis in endometrial polymorphonuclear leukocytes (PMN), Figure S5: Gating strategy for oxidative burst (OB) and intracellular proteolytic degradation (by DQ-ovalbumin assay, DQ-ova) in endometrial polymorphonuclear leukocytes (PMN); Figure S6: Polymorphonuclear leukocyte (PMN) functions oxidative burst (OB), phagocytosis (PC), and intracellular proteolytic degradation by DQ ovalbumin assay (DQ-ova) in blood or endometrial samples.
