# Supplementary material for: Flow Cytometric Assessment of the Viability and Functionality of Uterine Polymorphonuclear Leukocytes in Postpartum Dairy Cows
Source: Animals (Basel). 2021 Apr 10;11(4):1081. doi: 10.3390/ani11041081 (PMC8069149; doi:10.3390/ani11041081)
Supplement: Supplementary file 1 [file animals-11-01081-s001.pdf]

animals

Supplementary Materials: Flow Cytometric Assessment of the Viability and Functionality of Uterine Polymorphonuclear Leukocytes in Postpartum Dairy Cows

Leen Lietaer, Kristel Demeyere, Stijn Heirbaut, Evelyne Meyer, Geert Opsomer and Osvaldo Bogado Pascottini

Table S1. Fluorescent dyes and their excitation and emission characteristics.

Fluorescent dye	Channel	Excitation	Emission	Test
Alexa Fluor 647	APC	638 nm	660/20 nm	Viability assessment (secondary antibody)
Annexin-V-Fluos	FITC	488 nm	525/40 nm	Viability assessment (Apoptosis)
Propidium Iodide	PE	488 nm	585/42 nm	Viability assessment (Necrosis)
H2DCFDA	FITC	488 nm	525/40 nm	Oxidative Burst
FluoSpheres yellow-green	FITC	488 nm	525/40 nm	Phagocytosis
DQ ovalbumin	FITC	488 nm	525/40 nm	Intracellular proteolytic degradation

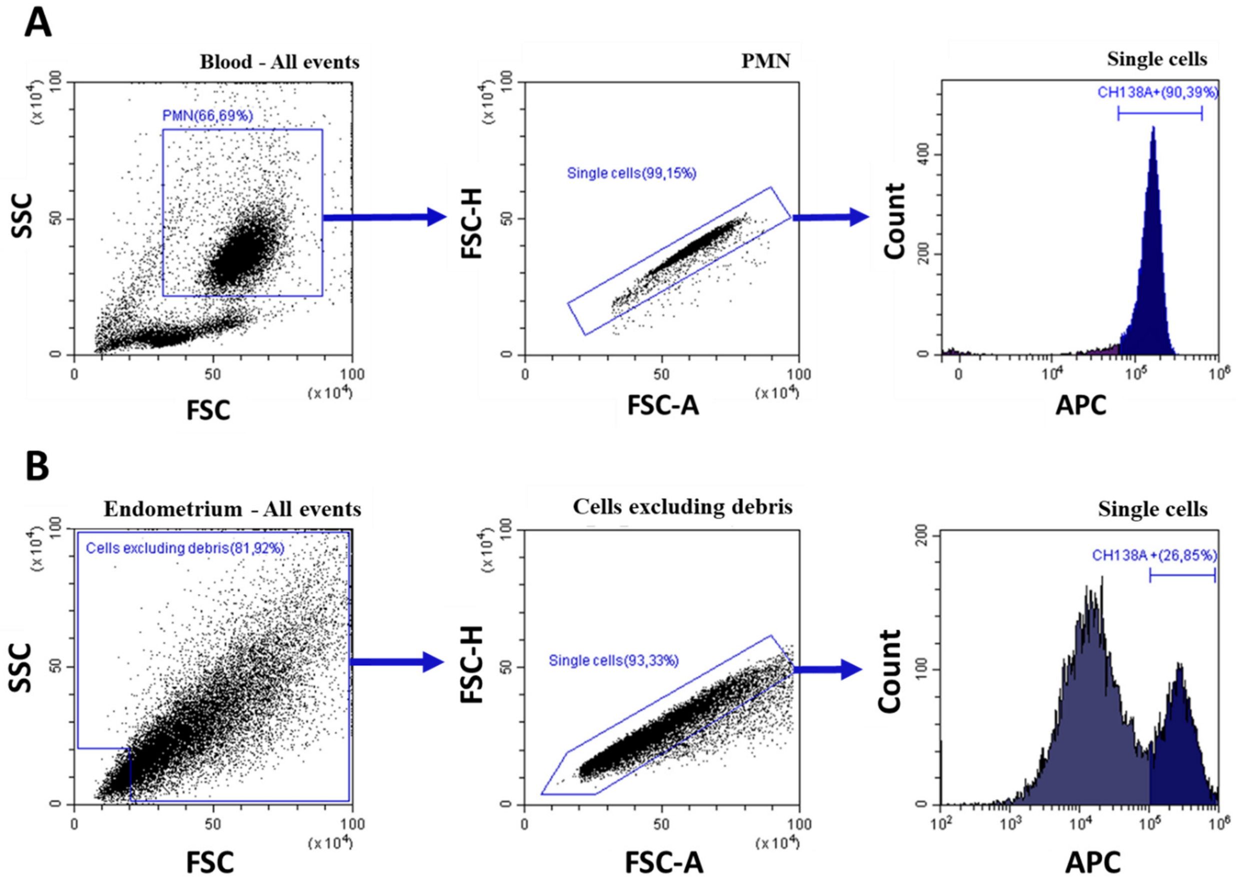

Figure S1. Gating strategy for flow cytometric assessment of viability in bovine circulating (**A**) versus endometrial (**B**) polymorphonuclear leukocytes (PMN). This figure shows representative data from one animal (9 DIM, 50% PMN on endometrial cytology).

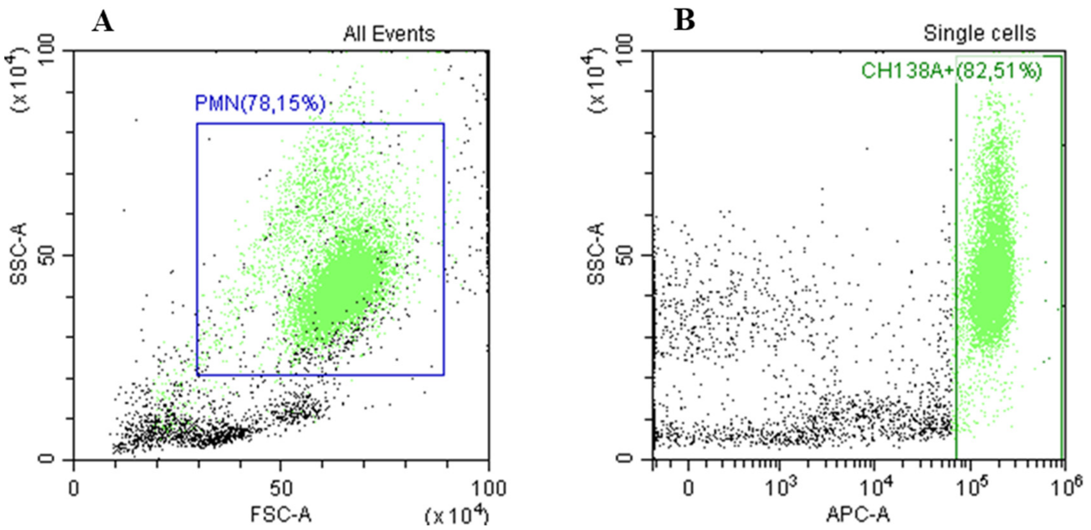

Figure S2. Flow cytometric identification of blood polymorphonuclear leukocytes (PMN) using CH138A. A) forward versus side scatter (**FSC vs. SSC**) plot of all events after PMN isolation from blood. The PMN gate is only illustrative. A single cell population was selected. B) Differentiation of isolated blood PMN based on CH138A x Alexa 647 positivity (CH138A = bovine granulocyte marker, APC channel).

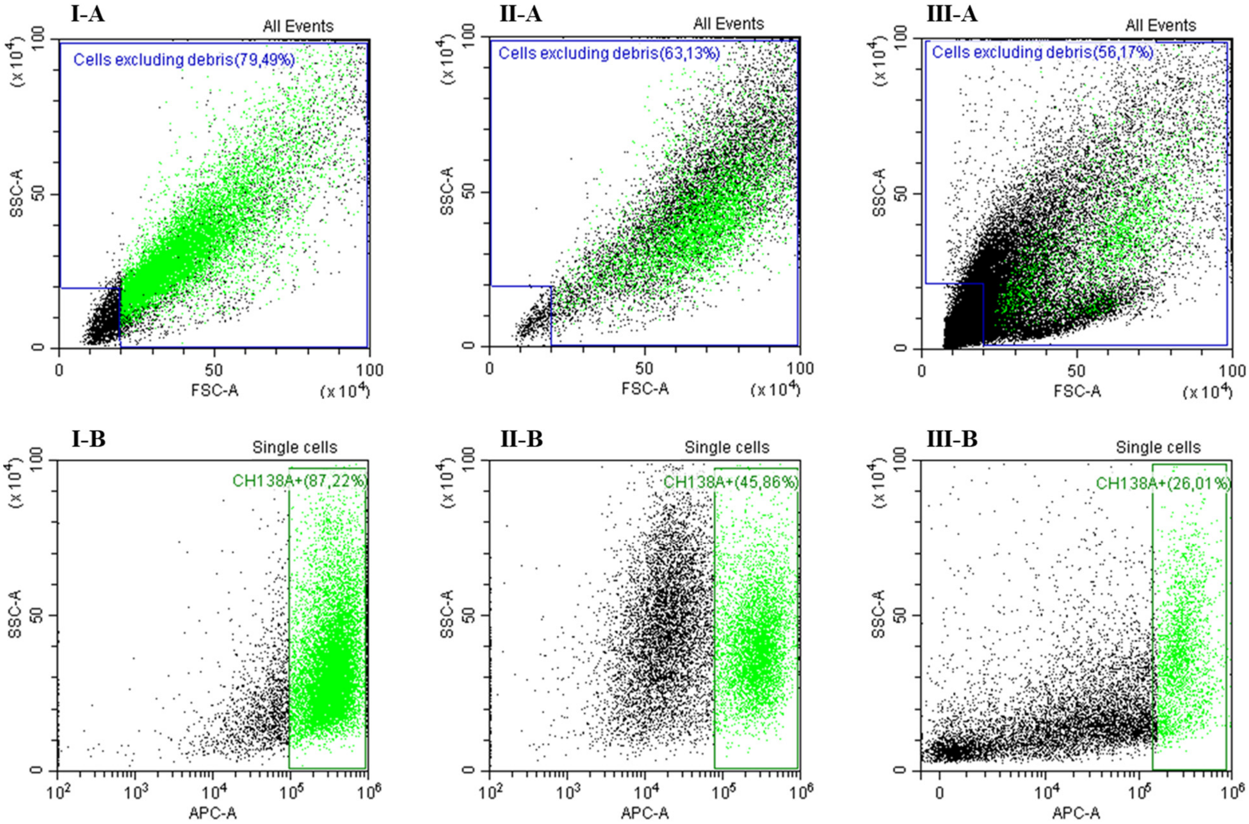

Figure S3. Flow cytometric identification of endometrial polymorphonuclear leukocytes (PMN) using CH138A. This figure shows representative samples (I and II) containing high proportion of PMN (9 DIM, 85% and 50% PMN, respectively, on endometrial cytology) and a representative sample (III) containing low proportion of PMN (36 DIM, 3% PMN on endometrial cytology). A) forward versus side scatter (**FSC vs. SSC**) plot of all events in endometrial cell suspensions. Excluding the events in the debris gate, a single cell population was selected. B) Differentiation of isolated endometrial PMN based on CH138A x Alexa 647 positivity (CH138A = bovine granulocyte marker, APC channel).

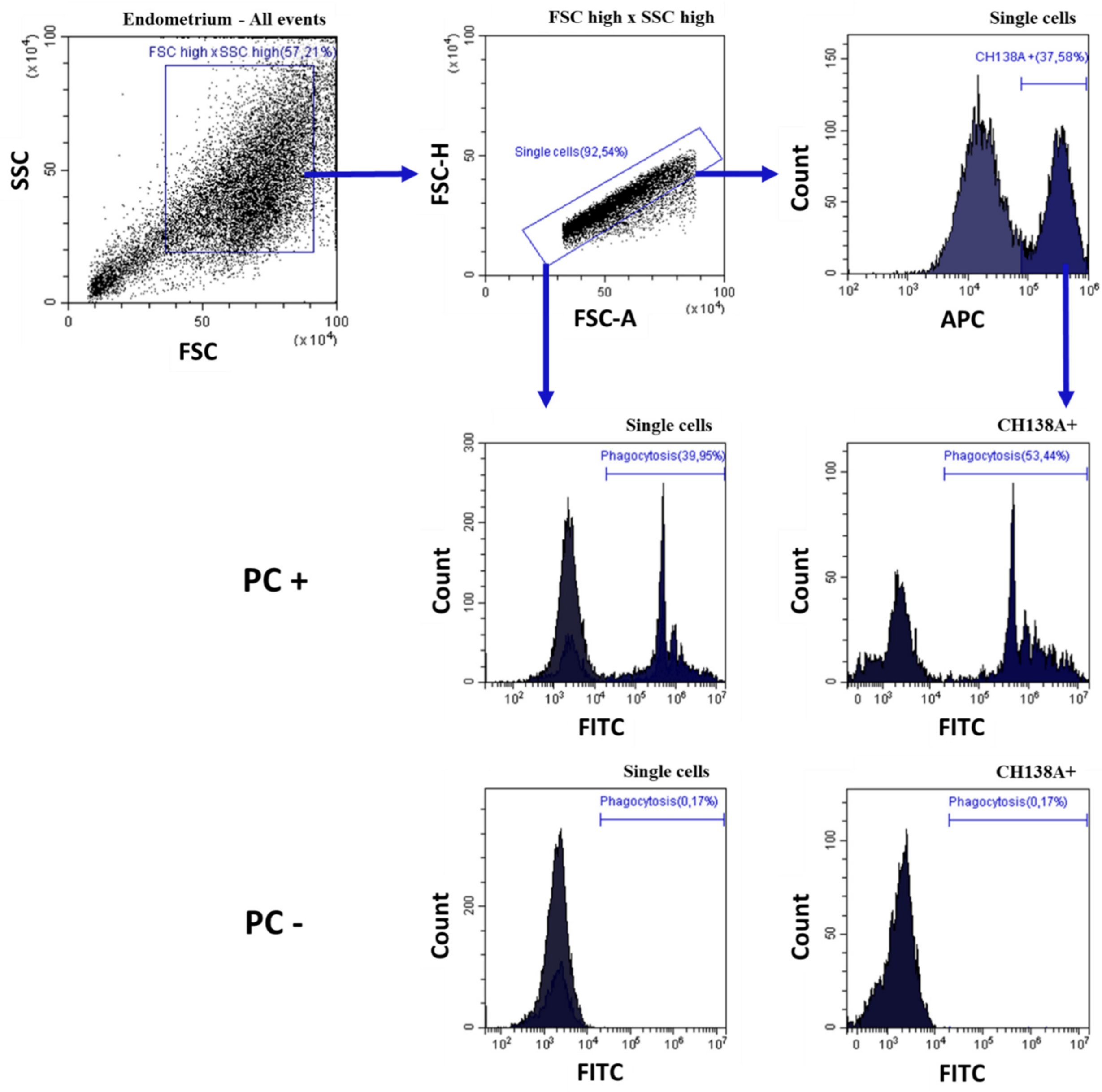

Figure S4. Gating strategy for phagocytosis in endometrial polymorphonuclear leukocytes (PMN). The PMN functionality tests (PC+) were performed in duplicate, one without (left) and one with (right) prior CH138A immunolabeling, each of them with their respective control group for immunofluorescence (PC-). Representative uterine sample from a dairy cow 9 days in milk, containing 50% PMN on endometrial cytology.

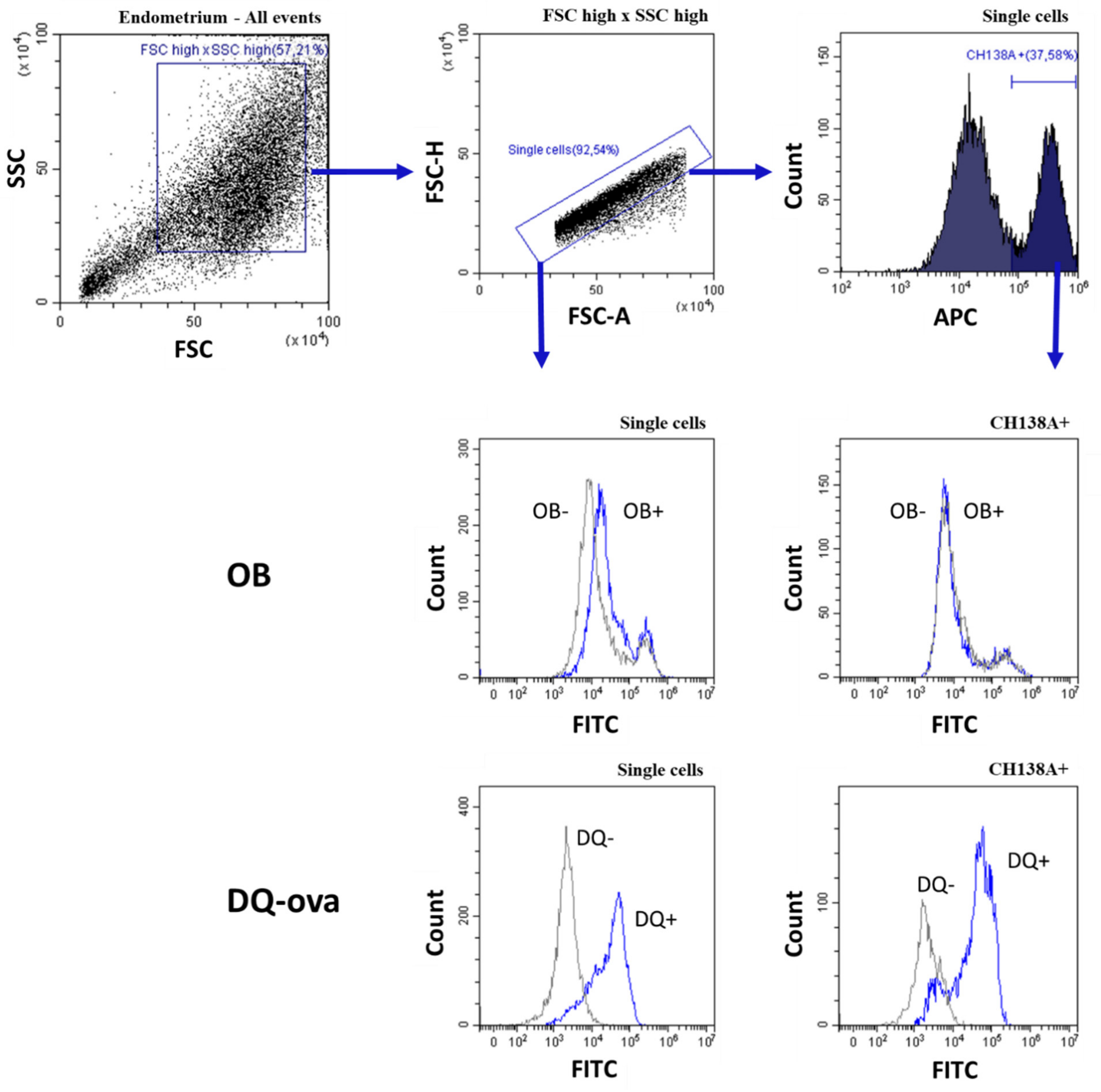

Figure S5. Gating strategy for oxidative burst (OB) and intracellular proteolytic degradation (by DQ-ovalbumin assay, DQ-ova) in endometrial polymorphonuclear leukocytes (PMN). The PMN functionality tests (OB+ and DQ+) were performed in duplicate, one without (left) and one with (right) prior CH138A immunolabeling, each of them with their respective control group for immunofluorescence (OB- and DQ-). Representative uterine sample from a dairy cow 9 days in milk, containing 50%PMN on endometrial cytology.

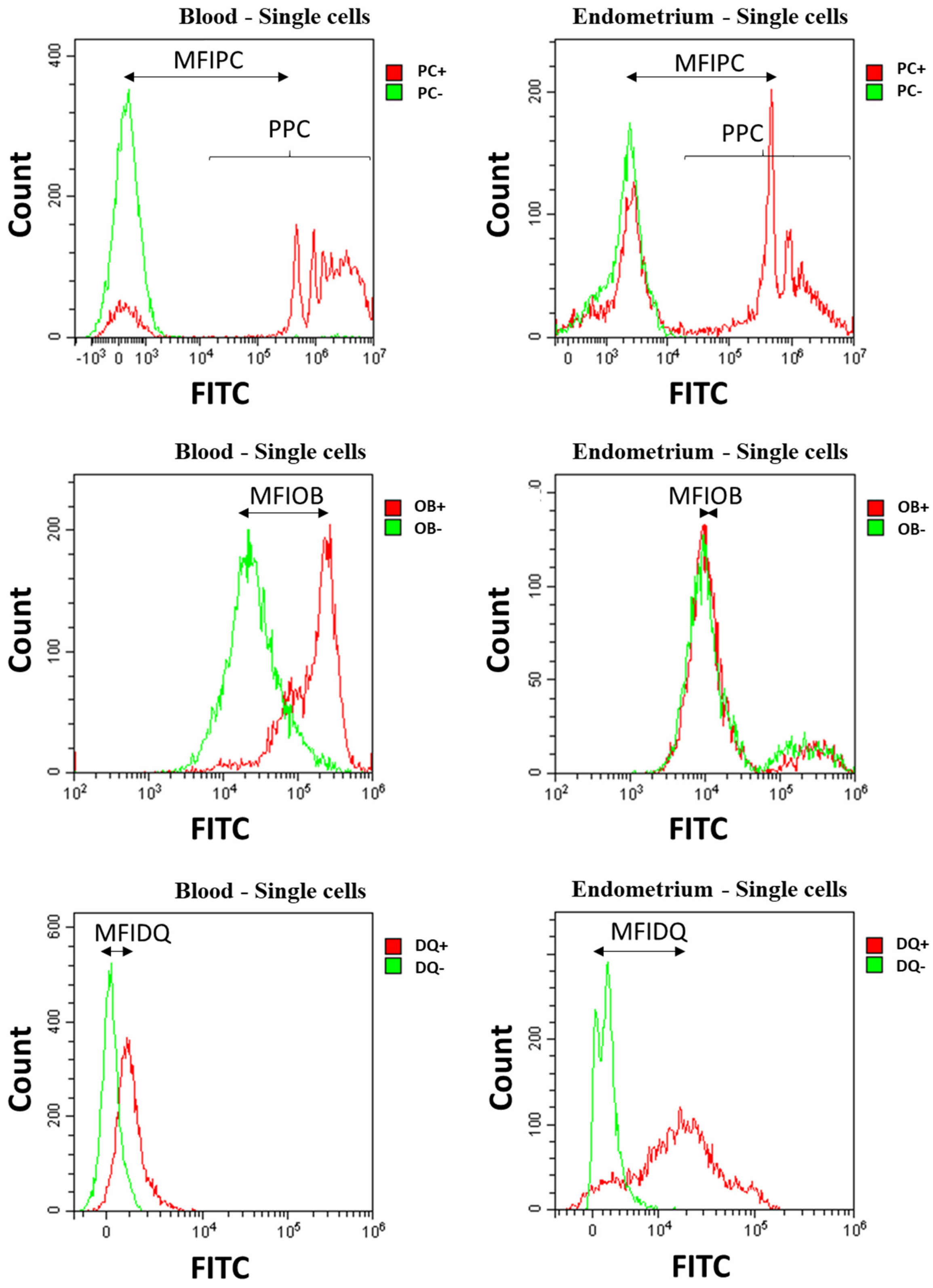

Figure S6. Polymorphonuclear leukocytes (PMN) functions oxidative burst (OB), phagocytosis (PC), and intracellular proteolytic degradation by DQ ovalbumin assay (DQ-ova) in blood or endometrial samples. The percentage of PMN that performs phagocytosis (PPC) and the difference in the median fluorescence intensity (MFI) of PMN that displayed OB (MFIOB), PC (MFIPC), or proteolytic degradation (MFIDQ) was calculated relative to the MFI of the respective autofluorescence control. Representative samples from a dairy cow 9 days in milk, containing 50% PMN on endometrial cytology.
